# Effectiveness of medical nutrition therapy in adolescents with type 1 diabetes: a systematic review

**DOI:** 10.1038/s41387-022-00201-7

**Published:** 2022-04-22

**Authors:** Minerva Granado-Casas, Ivan Solà, Marta Hernández, Marina Idalia Rojo-López, Josep Julve, Didac Mauricio

**Affiliations:** 1 Center for Biomedical Research on Diabetes and Associated Metabolic Diseases (CIBERDEM), Instituto de Salud Carlos III, Barcelona, Spain; 2 Department of Endocrinology and Nutrition, Hospital de la Santa Creu i Sant Pau & Sant Pau Biomedical Research Institute (IIB Sant Pau), Barcelona, Spain; 3 Institut d’Investigació Biomèdica Sant Pau (IIB Sant Pau), 08041 Barcelona, Spain; 4 Center for Biomedical Research on Epidemiology and Public Health (CIBERESP), Instituto de Salud Carlos III, Madrid, Spain; 5 Lleida Institute for Biomedical Research Dr. Pifarré Foundation IRBLleida, University of Lleida, Lleida, Spain; 6 Department of Endocrinology and Nutrition, University Hospital Arnau de Vilanova, Lleida, Spain; 7 Faculty of Medicine, University of Vic (UVIC/UCC), Vic, Spain

**Keywords:** Type 1 diabetes, Nutrition, Patient education

## Abstract

**BACKGROUND:**

Medical nutrition therapy (MNT) has an integral role in overall diabetes management. During adolescence, consideration of physiological and psychosocial changes is essential for implementing an optimal diabetes treatment.

**OBJECTIVES:**

Our aim was to identify, summarize, and interpret the published literature about MNT in adolescents with type 1 diabetes.

**METHODS:**

The Medline (PubMed) and EMBASE databases were searched from January 1959 to December 2021. The inclusion criteria were interventional studies with MNT in adolescents with type 1 diabetes with a disease duration over 1 year, including the following outcomes: dietary intake and daily eating patterns (assessed with validated tools, two or more 24 h dietary recall or 3-day dietary records), the diabetes self-management education and support (DSMES), glycemic control, lipid profile and body mass index (BMI). The exclusion criteria were studies without a control group (except for pre-post studies), the lack of randomization and those studies that assessed only a single nutrient, food or meal consumption, as well as reviews, and in-vitro/in-vivo studies. The risk of bias assessment was performed using the Cochrane risk-of-bias tool for randomized trials. A narrative synthesis was performed to present the results. The quality of evidence was assessed with the GRADE guidance.

**RESULTS:**

From a total of 5377 records, 12 intervention studies (9 RCT and 3 pre-post intervention studies) were included. The data were assessed in order to perform a meta-analysis; however, the studies were too heterogeneous. The studies showed conflicting results about the effectiveness of MNT on dietary pattern, DSMES, glycemic control, lipid profile and BMI.

**CONCLUSIONS:**

Clinical research studies on the effectiveness of MNT in adolescents with type 1 diabetes are scarce. The limited number of studies with a high risk of bias precludes establishing robust conclusions on this issue. Further research is warranted.

## Introduction

The American Diabetes Association (ADA) defines that lifestyle management is a cornerstone of diabetes care and includes diabetes self-management education and support (DSMES), medical nutrition therapy (MNT), physical activity, smoking cessation counseling, and psychosocial care [1]. For people with type 1 diabetes, MNT is a key point in the management of the disease and is also a component of DSMES. Carbohydrate counting and insulin management are mainly targeted to enable people with type 1 diabetes to self-manage the disease [2, 3]; furthermore, education about how to use fat and protein content to determine insulin dosing is recommended to improve glycemic control [2]. In addition, nutritional recommendations based on healthy dietary habits are included in the MNT of people with diabetes [4]. Nutrition therapy has an integral role in overall diabetes management, and people with diabetes should be actively engaged in education, self-management, and treatment planning with the healthcare team [1].

During adolescence, attention to family dynamics, developmental stages, and physiological changes related with sexual maturity are essential for implementing an optimal diabetes treatment [2]. Moreover, diabetes distress has been shown to be positively associated with disordered eating in adolescents with type 1 diabetes [5]. In addition, a poor adherence to nutritional recommendations has been observed in children and adolescents with type 1 diabetes [6]. Having a robust evidence-base regarding the effectiveness of MNT is important to inform healthcare professionals. The Academy of Nutrition and Dietetics published a systematic review to assess the effectiveness of MNT in adults with type 1 and type 2 diabetes so that healthcare professionals are knowledgeable about MNT for people with both types of diabetes [7, 8]. However, to our knowledge, a systematic review to assess the effectiveness of MNT in adolescents with type 1 diabetes has so far not been performed. Adolescents with type 1 diabetes have particular challenges: attention to family dynamics, developmental stages, and physiological changes related with sexual maturity are essential for implementing an optimal diabetes treatment [2]. Moreover, diabetes distress has been shown to be positively associated with disordered eating in adolescents with type 1 diabetes [5]. Furthermore, poor adherence to the nutritional recommendations has been observed in children and adolescents with type 1 diabetes. For this reason, in this systematic review, we aimed to identify, summarize, and interpret the published literature about MNT of adolescents with type 1 diabetes.

## Materials and methods

This systematic review followed the Preferred Reported Items for Systematic Review and Meta-analysis (PRISMA) checklist [9]. The protocol was registered with PROSPERO in April 2020 (ID: CRD42020162314).

### Search strategy

A systematic review was performed using the MEDLINE (PubMed) and EMBASE databases searched with a date range from January 1959 to December 2021. We included all published studies in peer-reviewed journals in the English language. The search strategy is available in Table S1. Mesh terms were included.

### Eligibility criteria

Studies were included in the systematic review if the study participants fulfilled the predefined inclusion criteria: studies that performed an intervention with MNT in adolescents diagnosed with type 1 diabetes with a disease duration of more than 1 year; adolescence was defined by the researchers of the included studies. Moreover, studies were included if the main outcomes were as follows: the dietary intake, which referred to the daily eating patterns of an individual (including specific foods, nutrients and calories consumed and relative quantities), estimated using validated food frequency questionnaires, two or more 24 h (h) dietary recall and a 3-day dietary record, at a minimum; the dietary pattern, which referred to the distribution and selection of daily food intake, estimated with validated quality dietary indexes; the DSMES including knowledge, skills, and abilities necessary for optimal diabetes self-care, incorporating the needs, goals, and life experiences of the person with diabetes; glycemic control including hypoglycemia and hyperglycemia defined according to the international consensus report; lipid profile and body mass index (BMI) [10].

### Study selection

Eligible study designs were randomized controlled trials (RCT), post-hoc analysis of interventional studies and pre-post interventional studies. Exclusion criteria were as follows: interventional studies without a control group, except for pre-post interventional studies; lack of randomization in controlled trials; studies that only assessed a single nutrient or consumption of a specific food or meal; the use of non-validated instruments, or the use of only one 24 h dietary recall to estimate dietary intake. Furthermore, the following types of articles were also excluded: reviews; studies performed with animals and in-vitro/in-vivo studies; studies without a defined outcome and papers with insufficient data to establish conclusions.

### Data extraction and risk of bias assessment

The selection of the studies was performed by three individual researchers. Studies were found to be eligible when two of the independent researchers agreed with the inclusion decision according to the inclusion and exclusion criteria. Nevertheless, in case of a discordant decision, this was discussed with a third researcher including a common thorough revision of inclusion/exclusion criteria of the systematic review. Data extraction was performed by a single researcher and checked by a second researcher; this included the following set of variables: first author’s surname, year of publication, journal, study design, country, sample size, study sample characteristics, age of participants, dietary assessment including instruments used, statistical methods and adjustment for potential confounders. Effect estimates from included studies was extracted to obtain data in terms of risk ratios (with 95% confidence intervals [CIs]) for dichotomous outcomes, and mean differences (with 95% CIs) for continuous outcomes. Disagreements were resolved by discussion with the corresponding author (DM).

The risk of bias assessment of the RCT was performed using the Cochrane risk-of-bias tool for randomized trials [11]. This tool classifies RCT according to the following domains: the randomization process, intended interventions, missing outcome data, measurement of the outcome and the selection of the reported results. RCT were considered to have a low risk of bias, uncertain and high risk [12]. In addition, non-randomized studies were assessed using the Risk of Bias in Non-Randomized Studies - of Interventions tool (ROBINS-I tool) [13]. This tool classifies the studies according to seven domains as follows: confounding, selection of participants into the study, classification of interventions, deviations from intended interventions, missing data, measurement of outcomes and selection of the reported results. The studies were classified according to the risk of bias as follows: low risk (the study was comparable with a well-performed randomized trial), moderate (the study provided sound evidence for a non-randomized study but could not be considered comparable with a well-performed randomized trial), serious risk (the study had some important problems), critical risk (the study was too problematic to provide any useful evidence and should not be included in the systematic review), and no information [14].

### Data synthesis

A summary table with the data extraction was performed including the risk of bias. Data analysis was performed with a sensibility analysis of the results taking into account the study design, and the risk of bias. A narrative synthesis of the findings of the review was performed according to the outcomes of interest. According to the GRADE guidance [15], the quality of evidence for primary outcomes was assessed as an expression of the confidence in the effect estimates obtained in the review through the assessment of risk of bias, directness of evidence, heterogeneity and precision of effect estimates.

## Results

**Fig. 1:**
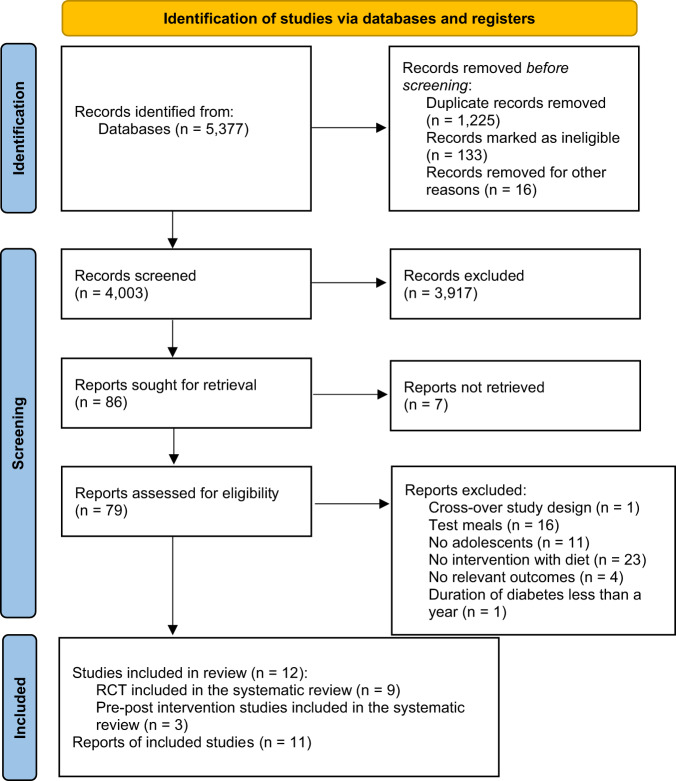
PRISMA flow diagram of the study selection. RCT randomized clinical trial.

A total of 5377 records were identified from the two databases search. Following the eligibility criteria, a total of 23 references reporting on 12 different studies were included in the systematic review. From these, 9 were RCT [16–24], 11 were post-hoc studies from these RCT [25–35], and 3 were pre-post intervention studies [36–38]. The PRISMA flow chart is provided in Fig. 1.

### Risk of bias of the included studies

A summary of the studies included in the systematic review is detailed in Table 1. A total of six randomized trials and the pre-post intervention studies showed a high risk of bias, while the other three RCT had uncertain risk. The details of the risk of bias assessment are described in Tables S2 and S3. Furthermore, a summary of included studies and their published reports is described in Table S4. The data were assessed in order to perform a meta-analysis; however, the studies were too heterogenous in terms of study duration, intervention type and the reported outcomes.

**Table 1:** Summary of the studies included in the systematic review.

Author	Sample size	Age of participants	Study sample characteristics	Intervention	Instruments	Outcomes	Statistical methods	Adjustment for confounders	Risk of Bias
*Randomized Clinical Trials*									
Dłużniak-Gołaska et al. [16]	*N* = 70 intervention with interactive methods and education *N* = 81 intervention with education without interactive methods	8–17 years	-Treated with insulin pumps. -No presence of chronic diseases whose require dietary modifications.	Interactive methods, i.e., quiz + multimedia application	NKS KomPAN	HbA1c Insulin dose Nutrition Knowledge BMI	*t*-test analysis of variance McNemar’s test Cochran–Mantel–Haenszel test	Not reported	High
Nansel et al. [17]	*N* = 66 intervention *N* = 70 control	8–16 years	-Daily insulin dose ≥0.5 units/kg -HbA1c 6.5–10.0% -3 or more injections daily or use of insulin pump -At least one clinic visit in the past year -Ability to communicate in English	Behavioral nutrition intervention to increase whole plant foods	3-day dietary records	HEI-2005 WPFD HbA1c	*t*-tests Pearson’s chi-square Permutation test	Not reported	High
Göksen et al. [18]	*N* = 52 intervention *N* = 32 control	7–18 years	-Before the study, traditional exchange-based meal plan -Glargine/detemir basal-bolus insulin regimens	Carbohydrate counting	Not reported	HbA1c Insulin dose Lipid profile BMI	Chi-square test *t*-test Mann–Whitney U-test ANOVA test	Not reported	High
Spiegel et al. [19]	*N* = 33 intervention *N* = 33 control	12–18 years	Participants using insulin to carbohydrate ratios for at least one meal a day.	Carbohydrate counting	3-day dietary record 32-item food frequency questionnaire	HbA1c	*t*-test Spearman correlation Fisher’s test	Group, age, sex and diabetes duration	High
Marquard et al. [20]	*N* = 9 OMD group *N* = 8 low-GI group	6–14 years	-Treatment with an intensive conventional insulin therapy. -No additional dietary restrictions. -Exclusion of other chronic diseases. -No medications that would affect appetite.	Two groups: - Optimized mixed diet - Flexible low-glycemic index diet	4-day dietary records	Nutritional Quality Index Macronutrient and micronutrient composition HbA1c (%) BMI	Wilcoxon signed-rank test Mann–Whitney U-test	Not reported	High
Gilbertson et al. [21]	*N* = 38 CHOx group *N* = 51 low-GI group	8–13 years	-Regular attendance at the clinic. -No additional dietary restrictions. -No immediate family members with diabetes. -No current medications that would affect the appetite. -Immediate family members with ability to read and write English.	Comparison between the CHOx with low-GI	3-day dietary records Quality of life questionnaire	HbA1c Hypoglycemia Hyperglycemia Insulin dose Dietary intake Quality of life	Multiple linear regression models Spearman’s correlation Logistic regression models Pearson’s chi-square test Wilcoxon’s rank-sum test	Energy intake, group, time-by-treatment interactions, HbA1c, insulin dose, hyperglycemia and hypoglycemia episodes	High
Donaghue et al. [22]	*N* = 12 intervention *N* = 11 control	14–21 years	Tanner stage 4–5 puberty.	MUFA-rich diet: 43% of fat, 20% of MUFA. Control group: 30% of fat, 55% of carbohydrate	4-day dietary records	HbA1c Insulin dose Lipid profile Body weight	*t*-test Wilcoxon rank-sum test Regression models	Not reported	Uncertain
Pichert et al. [23]	*N* = 69 participants	9–15 years	Adolescents participating in a diabetes camp	MNT	60-item test Dietary recalls	Nutrition knowledge skills	Covariance (inequities between the groups)	Not reported	Uncertain
Hackett et al. [24]	*N* = 119 families	Mean (SD): 11.4 (3.3) of the younger group, and 12.4 (3.6) of the older group.	Participants attending children’s diabetic clinic.	MNT	3-day dietary records	Dietary intake HbA1c	Analysis of covariance Linear models	Age, sex socioeconomic group, and duration of diabetes.	Uncertain
*Pre-post intervention*									
Marigliano et al. [36]	*N* = 25 participants	7–14 years	-Treatment with continuous subcutaneous insulin infusion (CSII) for at least 1 year. -White race. -No presence of retinopathy or microalbuminuria. -No presence of other autoimmune diseases.	Carbohydrate counting with standard nutritional education	4-day dietary records	HbA1c Insulin dose Dietary intake BMI Lipid profile	*t*-test	Not reported	Serious
Cadario et al. [37]	*N* = 64 participants	13–19 years	-Intensive treatment with continuous insulin injection or multiple daily injections. -HbA1c < 7.5 for adolescents and young adults (13–19 years) -No presence of nephropathy, abnormal liver or thyroid function. -No treatment for dyslipidemia. -No celiac disease.	Nutritional education according to the ADA recommendations	Dietary records Food frequency questionnaire	HbA1c Insulin dose Dietary intake BMI Lipid profile	Log transformed non-Gaussian data Chi-square test Fisher’s test McNemar’s test ANOVA Bonferroni test Wilcoxon and Mann-Whitney U-test	Not reported	Serious
Lorini et al. [38]	*N* = 36 participants	9–21 years	-Treated with twice daily administration insulin. -Visited at pediatric clinic every 2 months.	Intensive MNT	3-day dietary records	Dietary intake Insulin dose HbA1c Lipid profile	*t*-test	Not reported	No information

*ADA* American Diabetes Association, *BMI* body mass index, *CHOx* traditional carbohydrate-exchange dietary advice, *HbA1c* glycated hemoglobin, *HEI-2005* Healthy Eating Index-2005, *low-GI* low-glycemic index diet, *MNT* medical nutrition therapy, *MUFA* monounsaturated fatty acids, *OMD* optimized mixed diet.

### Effect of medical nutrition therapy on the dietary intake and diabetes self-management education

Studies that assessed the effectiveness of MNT for improving dietary pattern and DSMES are detailed in Table 2. A total of five RCT, six post-hoc from RCT and three pre-post intervention studies assessed these interventions. A RCT performed in Poland with a sample of 151 adolescents with type 1 diabetes found that an educative intervention with multimedia methods based on the ADA recommendations improved the nutrition knowledge and the dietary quality index of the participants [16]. Nansel et al. [17] demonstrated that a family-based behavioral intervention to increase whole plant food intake during an 18-month period increased the adherence to a healthy eating pattern in adolescents with type 1 diabetes compared to usual care (i.e., no dietary advice). Dietary resemblance (i.e., the diet of the child resembles the diet of the parent) was stronger in the intervention group for the outcome of whole plant food density (WPFD) [26], while an intervention effect on diet quality was only positive for a subgroup of picky eaters [28].

**Table 2:** Characteristics of the studies that assessed the effectiveness of the Medical Nutrition Therapy to improve dietary pattern and diabetes self-management education and support in adolescents with type 1 diabetes.

Author	Sample size	Age of participants	Intervention	Outcomes	Findings
*Randomized Clinical Trials*					
Dłużniak-Gołaska et al. [16]	*N* = 70 intervention with interactive methods and education (group E) *N* = 81 intervention with education without interactive methods (group C)	8–17 years	Interactive methods, i.e., quiz + multimedia application	Nutrition Knowledge	Nutrition Knowledge: Index of healthy diet: mean change (95% CI): −2.98 (−5.10;−0.86), *p* < 0.01 in the group C. Total NKS: mean change (95% CI): 3.00 (1.73;4.27), *p* < 0.001 in the group C, and 3.70 (2.56;4.84), *p* < 0.001 in the group E; *p* = 0.422 for the intergroup difference after 6 months.
Nansel et al. [17]	*N* = 66 intervention *N* = 70 control	8–16 years	Behavioral nutrition intervention to increase whole plant foods	HEI-2005 WPFD	HEI-2005 (mean (SE) = 64.6 (2.0) intervention group vs. 57.4 (1.6) control group, *p* = 0.015). WPFD (mean (SE) = 2.2 (0.1) intervention group vs. 1.7 (0.1) control group, *p* = 0.004)
Marquard et al. [20]	*N* = 9 OMD group *N* = 8 low-GI group	6–14 years	Two groups: - Optimized mixed diet - Flexible low-glycemic index diet	Nutritional Quality Index Macronutrient and micronutrient composition	OMD group (mean (SD)): Energy intake (Kcal) = 1767 (300) at baseline and 1532 (386) at follow-up; *p* = 0.05 Carbohydrate (g/day) = 214 (34) at baseline and 191 (62) at follow-up; *p* = 0.17 Total fat (g/day) = 72 (20) at baseline and 58 (15) at follow-up; *p* = 0.05 Protein (g/day) = 60 (10) at baseline and 57 (12) at follow-up; *p* = 0.09 Nutritional Quality Index = 79.5 (10.0) at baseline and 75.9 (14.0) at follow-up; *p* = 0.68 Low-GI group (mean (SD)): Energy intake (Kcal/day) = 1847.0 (281.0) at baseline and 1675.0 (203.0) at follow-up; *p* = 0.13. Carbohydrate (g/day): 236.0 (46.0) at baseline and 197.0 (31.0) at follow-up; *p* = 0.04 Total fat (g/day): 70.0 (19.0) at baseline and 70.0 (12.0) at follow-up; *p* = 0.99 Protein (g/day): 64.0 (16.0) at baseline and 59.0 (13.0) at follow-up; *p* = 0.17 Nutritional Quality Index = 79.5 (13.0) at baseline and 76.5 (10.0) at follow-up; *p* = 0.50
Pichert et al. [23]	*N* = 69 participants	9–15 years	MNT	Nutrition knowledge skills	All skills improved (*p* < 0.01) over time, achieving the primary goal of improving campers’ nutrition knowledge skills.
Hackett et al. [24]	*N* = 119 families	Mean (SD): 11.4 (3.3) of the younger group, and 12.4 (3.6) of the older group.	MNT	Dietary intake	Changes in diet were not significant.
*Post-hoc from RCT*					
Lipsky et al. [26]	*N* = 66 intervention *N* = 70 control	8–16 years	Behavioral nutrition intervention to increase whole plant foods	HEI-2005 WPFD Family meal frequency	Associations of parent-child diet quality by treatment assignation (control group as reference): (β (SE) = 1.57 (1.32) *p* = 0.23 for aHEI, and 0.21 (0.12), *p* = 0.08 for WPFD. Diet quality resemblance at final study: HEI (β (SE) = 0.29 (0.11), *p* = 0.009) of the intervention group WPFD (β (SE) = 0.33 (0.09), *p* < 0.001) of the intervention group
Nansel et al. [29]	*N* = 66 intervention *N* = 70 control	8–16 years	Behavioral nutrition intervention to increase whole plant foods	HEI-2005 WPFD Pickiness subscale of the Child Feeding Questionnaire	The intervention effect on diet quality was positive for picky eaters only (WPFD *p* < 0.001; HEI *p* = 0.04).
Eisenberg-Colman et al. [30]	*N* = 42 intervention *N* = 48 control	8–16 years	Behavioral nutrition intervention to increase whole plant foods	Adherence to diabetes management tasks Disordered eating behaviors (DEB) DEPS-R	Intervention on DEB: β (SE) = 0.00 (0.00), *p* = 0.84 DEPS-R vs. diabetes management: β (SE) = −0.20 (0.09), *p* = 0.03
Eisenberg et al. [31]	*N* = 66 intervention *N* = 70 control	8–16 years	Behavioral nutrition intervention to increase whole plant foods	Dietary intake Whole Plant Food Density (WPFD) Self-Efficacy for Healthy Eating Outcome Expectations for Healthy Eating Barriers to Healthy Eating Treatment Self-Regulation Parent Nutrition Knowledge	Parent self-efficacy vs. WPFD (*β* = 0,21, *p* = 0.02) Autonomous motivation vs. WPFD (*β* = 0.17, *p* = 0.002) Nutrition knowledge vs. WPFD (*β* = 0.01, *p* = 0.03) Barriers to Healthy Eating vs. WPFD (*β* = −0.19, *p* = 0.02) Negative Outcomes Expectation vs. WPFD (*β* = −0.20, *p* = 0.008) Parent positive outcome expectations vs. WPFD (*β* = 0.07, *p* = 0.45) Controlled motivation vs. WPFD (*β* = −0.03, *p* = 0.56)
Gilbertson et al. [27]	*N* = 38 CHOx group *N* = 51 low-GI group	8–13 years	Comparison between the CHOx with low-GI	Macronutrient intake Carbohydrate food sources Carbohydrate distribution of meals and snacks	Energy intake (MJ/day): CHOx group vs. low-GI group (mean (SD) = 9.1 (1.7) vs. 9.3 (1.4), respectively). Protein (%): CHOx group vs. low-GI group (mean (SD) = 16.3 (1.9) vs. 16.3 (4.1), respectively). Total fat (%): CHOx group vs. low-GI group (mean (SD) = 35.3 (5.4) vs. 36.2 (6.3), respectively). Carbohydrate (%): CHOx group vs. low-GI group (mean (SD) = 48.3 (5.2) vs. 47.7 (6.2), respectively). Sugars (%): CHOx group vs. low-GI group (mean (SD) = 17.3 (5.8) vs. 18.8 (5.2), respectively). Fiber (g/day): CHOx group vs. low-GI group (mean (SD) = 22.4 (4.1) vs. 23.0 (7.2), respectively). No differences in carbohydrate distribution meals between bot groups.
*Pre-post intervention*					
Marigliano et al. [36]	*N* = 25 participants	7–14 years	Carbohydrate counting with standard nutritional education	Dietary intake	Energy intake (Kcal) (mean (SD) = 1595 (293.7) at baseline and 1766 (376.9) at follow-up, *p* < 0.01). Carbohydrates (%) (mean (SD) = 53.9 (4.6) at baseline and 56.7 (3.0) at follow-up, *p* < 0.01). Total fat (%) (mean (SD) = 30.8 (4.3) at baseline and 28.9 (2.7) at follow-up, *p* < 0.05). Protein (%) (mean (SD) = 15.0 (1.6) at baseline and 13.9 (1.7) at follow-up, *p* < 0.01).
Cadario et al. [37]	*N* = 64 participants	13–19 years	Nutritional education according to the American Diabetes Association recommendations	Dietary intake	Energy intake (Kcal/day) mean (SEM) = 1846.3 (43.8) at baseline and 1570.0 (36.6) at follow-up; p value was not significant. Carbohydrates (g/day) mean (SEM) = 238.2 (6.5) at baseline and 228.1 (5.5) at follow-up; *p* value was not significant. Total fat (g/day) mean (SEM) = 72.1 (2.5) at baseline and 49.1 (1.8) at follow-up; *p* < 0.05. Protein (g/day) mean (SEM) = 70.9 (1.5) at baseline and 68.2 (2.1) at follow-up; *p* value was not significant. Cholesterol (mg/day) mean (SEM) = 265.7 (9.1) at baseline and 94.1 (7.6) at follow-up; *p* < 0.01. Fiber (g/day) mean (SEM) = 18.5 (0.6) at baseline and 29.3 (1.1) at follow-up; *p* < 0.01.
Lorini et al. [38]	*N* = 36 participants	9–21 years	Intensive MNT	Dietary intake	Energy intake (Kcal/d) (mean (SD)): 2083 (554) vs. 1695 (581), *p* < 0.001. Total fat (%) (mean (SD)): 38.7 (6.2) vs. 34.3 (6.4), *p* < 0.01 Protein (%) (mean (SD)): 16.1 (2) vs. 15.4 (2), *p* was not significant. Carbohydrate (%) (mean (SD)): 44.8 (6.9) vs. 50 (8.2), *p* < 0.001. Carbohydrate-simple (g) (mean (SD)): 59.2 (23.8) vs. 65.2 (22.2), *p* < 0.05. Saturated fatty acid (g) (mean (SD)): 34.4 (9.6) vs. 27.3 (7.8), *p* < 0.001. PUFA (g) (mean (SD)): 12.1 (8.1) vs. 7.9 (3), *p* < 0.01. Fiber (g) (mean (SD)): 3.2 (1.6) vs. 3.8 (1.9), *p* < 0.05.

*BMI* body mass index, *CI* confidence interval, *CHOx* traditional carbohydrate-exchange dietary advice, *HbA1c* glycated hemoglobin, *HEI-2005* Healthy Eating Index-2005, *KomPAN* questionnaire for the study of views and dietary habits, *low-GI* low-glycemic index diet, *MNT* medical nutrition therapy, *NKS* Nutrition Knowledge Survey, *OMD* optimized mixed diet, *PUFA* polyunsaturated fatty acids, *SE* standard error, *SD* standard deviation, *WPFD* whole plant food density.

A pilot-study performed with a small sample of adolescents (*N* = 17) showed that an intervention with an optimized-mixed diet for 3 months (based on five meals per day with more than 50% of whole grain and low consumption of sweetened beverages) reduced protein intake [20]. Moreover, an intervention with a flexible low-glycemic diet (based on a list of avoid food consumption with low-glycemic index, free meals, whole grains and a poor consumption of beverages) reduced the carbohydrate intake [20]; however, the researchers did not find differences between both groups of intervention in terms of nutrient intake and Nutritional Quality Index. On the other hand, Hackett et al. [24] did not find changes in the dietary intake of adolescents and children that received a MNT based on the ADA nutritional recommendations. Moreover, Gilbertson et al. [35] did not find differences in reported nutrient intake between an intervention based on a low-glycemic index (low-GI) diet and a traditional carbohydrate-exchange diet.

In a pre-post intervention study, an intervention based on carbohydrate counting with nutritional recommendations by ADA was associated with an increase in carbohydrate intake and a reduced total fat and protein intake from baseline to 18-months of follow-up [36]. Moreover, MNT also based on the ADA nutritional recommendations, reduced the total fat and cholesterol intake, and increased the fiber consumption of adolescents in a 6-month pre-post intervention study [37]. In addition, Lorini et al. [38] observed a reduction of energy intake, total fat, polyunsaturated and saturated fatty acids, and an increased intake of carbohydrate and fiber after an intensive MNT based on nutritional guidelines for the general population.

In terms of DSMES, a post-hoc analyses of a RCT performed to assess the relationship between parent attitudes and youth diet quality, described no effect of the intervention on parent attitudes or beliefs [30]; however, a higher parent self-efficacy and autonomous motivation were positively associated with those youth with a higher adherence to a WPFD diet. Furthermore, a secondary data analysis from this RCT found that a behavioral intervention to improve dietary quality did not increase disordered eating behaviors in adolescents [29]; nevertheless, a greater adherence to diabetes self-management was associated with lower diabetes eating problems in adolescents with type 1 diabetes [29].

### Effect of medical nutrition therapy on glycemic control

The characteristics of the studies that reported the effect of a dietary intervention on glycemic control in adolescents with type 1 diabetes are shown in Table 3. Dłużniak-Gołaska et al. [16] found a significant reduction in glycated hemoglobin (HbA1c) concentrations after 3 months of treatment with educational materials plus interactive methods compared to a control group with educational methods alone. However, HbA1c was not reduced after 6 months from treatment initiation even though the dose of insulin was increased in the intervention group [16]. Hackett et al. [24] found an improved glycemic control in adolescents over age 11 years after MNT based on nutritional recommendations established in 1989. A RCT performed to assess the effect of an intervention with a WPFD did not find differences in HbA1c between the intervention and control groups [17]; however, a post-hoc analysis of this RCT described that an optimal glycemic control was associated with a healthier eating pattern (measured with the Healthy Eating Index-2005) and a higher adherence to a WPFD diet [34]. In addition, a RCT and a pre-post interventional study with MNT based on carbohydrate counting found a positive effect on the glycemic control of adolescents with type 1 diabetes [18, 36]; however, the dose of insulin was not modified with the intervention. A RCT performed to compare a traditional carbohydrate-exchange diet with a low-GI diet found a slight improvement of HbA1c with the carbohydrate-exchange diet [21]. On the other hand, Cadario et al. [37] did not find changes in glycemic control of the study participants even though an increased dose of insulin was observed with a nutritional intervention based on the ADA recommendations. Furthermore, the other studies did not observe any effect of dietary intervention on glycemic control in adolescents with type 1 diabetes [19, 20, 22, 38].

**Table 3:** Characteristics of the studies related to the effectiveness of Medical Nutrition Therapy on glycemic control in adolescents with type 1 diabetes.

Author	Sample size	Age of participants	Intervention	Outcomes	Findings
*Randomized Clinical Trials*					
Dłużniak-Gołaska et al. [16]	*N* = 70 intervention with interactive methods and education (group E) *N* = 81 intervention with education without interactive methods (group C)	8–17 years	Interactive methods, i.e., quiz + multimedia application	HbA1c Insulin dose	HbA1c (%): mean change (95% CI): − 0.47 (−0.77; −0.17), *P* < 0.01 in the group E; *p* = 0.038 for the intergroup difference after 3 months. However, no significant changes within groups after 6 months of treatment. Insulin dose (U/Kg BW/d): mean change (95% CI): 0.07 (0.02;0.13), *p* < 0.05 in the group E.
Nansel et al. [17]	*N* = 66 intervention *N* = 70 control	8–16 years	Behavioral nutrition intervention to increase whole plant foods	HbA1c	HbA1c (%) (mean (SE) = 8.3 (0.1) intervention group vs. 8.2 (0.1) control group); *p* value was not different between both groups.
Göksen et al. [18]	*N* = 52 intervention *N* = 32 control	7–18 years	Carbohydrate counting	HbA1c Insulin dose	HbA1c (%) (mean (SD) = 8.76 (1.77) for the control and 7.87 (1.38) for the intervention group, *p* = 0.010). Insulin dose (U/Kg/d) (mean (SD) = 1.02 (0.31) for the controls and 1.01 (0.28) for the intervention group, *p* = 0.643).
Spiegel et al. [19]	*N* = 33 intervention *N* = 33 control	12–18 years	Carbohydrate counting	HbA1c	Mean (SD) = −0.19 (0.12) for intervention group and −0.08 (0.11) for the control group; *p* = 0.49.
Marquard et al. [20]	*N* = 9 OMD group *N* = 8 low-GI group	6–14 years	Two groups: - Optimized mixed diet - Flexible low-glycemic index diet	HbA1c	OMD group (mean (SD)): HbA1c (%) = 7.4 (0.6) at baseline and 7.3 (0.5) at follow-up; *p* = 0.44 Low-GI group (mean (SD)): HbA1c (%) = 7.0 (0.5) at baseline and 6.9 (0.5) at follow-up; *p* = 0.61
Gilbertson et al. [21]	*N* = 38 CHOx group *N* = 51 low-GI group	8–13 years	Comparison between the CHOx with low-GI	HbA1c Incidence of hypoglycemia and hyperglycemia Insulin dose	HbA1c (%): CHOx group vs. low-GI group (mean (SD) = 8.6 (1.4) vs. 8.0 (1.0), respectively; *p* = 0.05). Insulin dose (UI/Kg): CHOx group vs. low-GI group (mean (SD) = 1.0 (0.3) vs. 1.1 (0.3), respectively; *p* = 0.87). Episodes of hyperglycemia (mean number per month): CHOx group vs. low-GI group (mean (SD) = 16.8 (11.8) vs. 11.2 (9.8), respectively; *p* = 0.06). Episodes of hypoglycemia (mean number per month): CHOx group vs. low-GI group (mean (SD) = 5.8 (5.5) vs. 6.9 (6.8), respectively; *p* = 0.37).
Donaghue et al. [22]	*N* = 12 intervention *N* = 11 control	14–21 years	MUFA-rich diet: 43% of fat, 20% of MUFA. Control group: 30% of fat, 55% of carbohydrate	HbA1c Insulin dose	HbA1c (%) (median [IQR] = 8.8 [8.2–9.5] for the intervention group vs. 9.3 [8.0–10.4] for the control group; *p* = 0.80). Insulin dose (UI/Kg) (mean (SD) = 1.0 (0.2) for the intervention group vs. 0.9 (0.2) for the control group; *p* = 0.70).
Hackett et al. [24]	*N* = 119 families	Mean (SD): 11.4 (3.3) of the younger group, and 12.4 (3.6) of the older group.	MNT	HbA1c	In adolescents under 11 years, no changes in HbA1c were observed. In adolescents over 11 years, an improved HbA1c was observed (*p* = 0.02).
*Post-hoc from RCT*					
Nansel et al. [35]	*N* = 66 intervention *N* = 70 control	8–16 years	Behavioral nutrition intervention to increase whole plant foods	HbA1c 1.5-AG BG values MAGE	HbA1c (%) vs. HEI-2005 and WPFD: *β* (SE) = 0.003 (0.003), *p* = 0.36 and −0.03 (0.03), *p* = 0.27, respectively. 1.5-AG (µg/mL) vs. HEI-005 and WPFD: (*β* (SE) = 0.004 (0.008), *p* = 0.62 and 0.16 (0.08), *p* = 0.05, respectively). Mean of BG values vs. HEI-2005 and WPFD: β (SE) = −0.41 (0.15), *p* = 0.005 and −4.35 (1.60), *p* = 0.006, respectively. MAGE vs. HEI-2005 and WPFD (β (SE) = −0.59 (0.20), *p* = 0.003 and −6.74 (2.14), *p* = 0.002, respectively).
*Pre-post intervention*					
Marigliano et al. [36]	*N* = 25 participants	7–14 years	Carbohydrate counting with standard nutritional education	HbA1c Insulin dose	HbA1c (%) (mean (SD) = 8.50 (0.77) at baseline and 7.92 (0.74) at follow-up, *p* < 0.001). Insulin dose (IU/Kg/d) (mean (SD) = 0.80 (0.21) at baseline and 0.78 (0.18) at follow-up, *p* = 0.411).
Cadario et al. [37]	*N* = 64 participants	13–19 years	Nutritional education according to the American Diabetes Association recommendations	HbA1c Insulin dose	HbA1c (%) mean (SEM) = 8.3 (0.1) at baseline and 8.2 (0.1) at follow-up; *p* was not statistically significant. Insulin dose (UI/day): mean (SEM) = 44.8 (1.9) at baseline and 50.6 (1.8) at follow-up; *p* < 0.001.
Lorini et al. [38]	*N* = 36 participants	9–21 years	Intensive MNT	Insulin dose HbA1c Lipid profile	HbA1c (%) (mean (SD)): 10.6 (2.6) vs. 10.9 (2.6); *p* was not statistically significant. Insulin dose (U/Kg/d) (mean (SD)): 0.98 (0.33) vs. 0.98 (0.34); *p* was not statistically significant.

*BG* blood glucose, *BMI* body mass index, *CHOx* traditional carbohydrate-exchange dietary advice, *CI* confidence interval, *HbA1c* glycated hemoglobin, *HDL* high-density lipoprotein,*HEI-2005* Healthy Eating Index-2005, *IQR* interquartile range, *KomPAN* questionnaire for the study of views and dietary habits, *LDL* low-density lipoprotein, *low-GI* flexible low-GI dietary advice, *MAGE* mean amplitude of glycemic excursions, *MNT* medical nutrition therapy, *MUFA* monounsaturated fatty acid, *NKS* Nutrition Knowledge Survey, *OMD* optimized mixed diet, *1.5-AG* 1.5-Anhydroglucitol, *SE* standard error, *SD* standard deviation, *WPFD* whole plant food density.

### Effect of medical nutrition therapy on lipid profile and body mass index

The effectiveness of MNT on cardiovascular risk factors, i.e., BMI and lipid profile, in adolescents with type 1 diabetes is shown in Table 4. Only two RCT with their post-hoc studies and one pre-post intervention study assessed this issue [16, 18, 27, 32, 38]. Dłużniak-Gołaska et al. [16] found that trial participants had an increased BMI in both the control group (education alone) and intervention groups (education with an interactive intervention with multimedia applications) after 6 months from treatment initiation; no between-group differences were observed. On the other hand, a post-hoc analyses from a randomized controlled behavioral nutrition intervention trial to assess the associations of BMI and body composition with cardiovascular risk factors did not find an intervention effect on cardiovascular risk factors after 18 months from treatment initiation [32].

**Table 4:** Studies that assessed the effectiveness of the Medical Nutrition Therapy on body mass index and lipid profile in adolescents with type 1 diabetes.

Author	Sample size	Age of participants	Intervention	Outcomes	Findings
*Randomized Clinical Trials*					
Dłużniak-Gołaska et al. [16]	*N* = 70 intervention with interactive methods and education (group E) *N* = 81 intervention with education without interactive methods (group C)	8–17 years	Interactive methods, i.e., quiz + multimedia application	BMI	SDS-BMI (Kg/m^2^): mean change (95% CI) after 6 months: 0.14 (0.06;0.21), *p* < 0.001 in the control group and 0.19 (0.09;0.28), *p* < 0.001 in the intervention group. No differences between both groups after 6 months of treatment (*p* = 0.370).
Göksen et al. [18]	*N* = 52 intervention *N* = 32 control	7–18 years	Carbohydrate counting	Lipid profile	No association was observed between the treatment assignment and lipid profile.
*Post-hoc from RCT*					
Sanjeevi et al. [28]	*N* = 66 intervention *N* = 70 control	8–16 years	Behavioral nutrition intervention to increase whole plant foods	Lipid profile	HEI-2005: Intervention vs. control (mean (SD) = 45.33 (12.44) vs. 46.73 (11.01), *p* = 0.49; respectively). HEI-2005 vs. Lipid profile (TG, TC, HDL and LDL): no association. Whole grains vs. TC, HDL and LDL (β (SE) = −4.60 (2.05), *p* = 0.03; −1.98 (0.09), *p* = 0.046, and −3.42 (1.92), *p* = 0.08; respectively). Refined grains vs. TG and LDL (β (SE) = −0.02 (0.01), *p* = 0.08, and 1.72 (0.98), *p* = 0.08; respectively). Sodium vs. LDL (β (SE) = 5.38 (3.10), *p* = 0.08). Added sugars (%, Kcal) vs. TG (β (SE) = 0.004 (0.002), *p* = 0.04). Saturated fat (%, Kcal) vs. HDL (0.34 (0.17), *p* = 0.04).
Lipsky et al. [33]	*N* = 66 intervention *N* = 70 control	8–16 years	Behavioral nutrition intervention to increase whole plant foods	BMI Lipid profile	Body fat was associated with TG (β (SE) = 0.01 (0.01), *p* = 0.006) and LDL (β (SE) = 0.11 (0.03), *p* = 0.001), and HDL (β (SE) = −0.06 (0.00), *p* = 0.04). BMI was associated with TG (β (SE) = 0.05 (0.01), *p* < 0.001) and HDL (−0.02 (0.01); *p* < 0.001). There was no intervention effect on cardiovascular risk factors.
*Pre-post intervention*					
Lorini et al. [38]	*N* = 36 participants	9–21 years	Intensive MNT	Lipid profile	HDL (mg/dl) (mean (SD)): 50.8 (15.8) vs. 58.7 (14.8), *p* < 0.01. LDL (mg/dl) (mean (SD)): 117.9 (50.5) vs. 106.8 (53.5), *p* < 0.05. TC and TG were not different.

*BMI* body mass index, *TC* total cholesterol, *HDL* high-density lipoprotein, *HEI-2005* Healthy Eating Index-2005, *group E* experimental group, *group C* control group, *LDL* low-density lipoprotein, *MNT* medical nutrition therapy, *SDS-BMI* standard deviation score-body mass index, *TG* triglycerides, *WPFD* whole plant food density.

In terms of lipid profile, a post-hoc analyses from an RCT designed to examine the association of cardiovascular biomarkers with dietary quality diet observed that a healthy eating pattern was not associated with lipid profile [27]. However, the intake of whole grain was inversely related with total cholesterol and high-density lipoprotein (HDL) cholesterol; moreover, added sugars and saturated fat were positively associated with triglycerides and HDL cholesterol, respectively [27]. On the other hand, a RCT to assess the effect of carbohydrate counting on serum lipid levels did not find differences between the intervention and control group during 2 years of follow-up [18]; nevertheless, HDL cholesterol was higher in the carbohydrate counting group during the study follow-up. Finally, Lorini et al. [38] found a reduction of HDL and LDL cholesterol levels after 3 months of intensive MNT in a pre-post intervention study.

## Discussion

Only 12 interventional studies were identified in this systematic review, demonstrating the paucity of evidence about the effectiveness of MNT in adolescents with type 1 diabetes. In addition, the included studies had a small sample size and a high risk of bias, further contributing to a lack of a robust evidence base. Furthermore, results could not be meta-analyzed because the study methods, i.e., duration of the study, intervention type and data, were not comparable. In addition, this review included a number of post-hoc analysis and publications derived of a single trial [17].

According to this review, an intervention with MNT based on carbohydrate counting as well as with multimedia methods, whole plant food intake or nutritional recommendations, has a positive impact on the dietary pattern of the adolescents with type 1 diabetes. These benefits on dietary intake were shown as a reduction of total fat and protein intake, an increased adherence to a healthy eating pattern, a higher parent-child WPFD diet, and a high intake of fiber. However, inconsistent results in carbohydrate intake were reported, with some studies observing an increased carbohydrate intake with standard nutritional recommendations, whereas others found a reduced carbohydrate intake with an intervention based on a low-GI diet [20, 36, 38]. These contradictory results could be due to different study methods, type of interventions and duration of the study. Moreover, there may have been differences in the study participants, while even the fact of participating in an RCT can impact on the lifestyle of the study participants, changing their dietary habits and daily routines to different extents. Overall, there was a lack of robust evidence about the effects of MNT on dietary patterns. This is in line with a recent systematic review that reported a lack of evidence to assess the effectiveness of technology-based interventions on dietary habits in children and young people with type 1 diabetes [39].

In terms of DSMES, only two post-hoc analyses from a RCT addressed this issue [29, 30]. The authors found that a higher adherence to a WPFD diet was associated with a higher parent self-efficacy and motivation [30]. Furthermore, adolescents with a healthy eating diet showed a lower presence of disordered eating behaviors. A cross-sectional study found that disordered eating behaviors were associated with a poorer diet quality in adolescents with type 1 diabetes [40]. Of note, another cross-sectional study observed that adolescents with a diabetes duration of 5 years or more had less diabetes care activities compared with individuals with shorter diabetes duration [41]. A review performed to assess the effects of carbohydrate-restricted diets in youth with type 1 diabetes described that the relationship between carbohydrate counting therapies and quality of life had still not been adequately assessed among youth with type 1 diabetes [42]. However, this review suggested that restrictive dietary practices may be related with the presence of disordered eating behaviors in adolescents with type 1 diabetes [42]. A systematic review performed to assess the relationship between psychological factors and diabetes self-management observed that a greater adherence to the diet had stronger effects on cognitive and emotional variables [43]; these included greater motivation, dietary self-efficacy, perceived support for autonomy and from family, and stronger beliefs with the effectiveness of behavior for diabetes and complications. In addition, diabetes-specific disordered eating behaviors are more frequent in girls in comparison with boys [44, 45]. Insulin under-dosing, intentional vomiting, feeling of losing control over food, a short-term weight loss (over 6 kg), and body dissatisfaction are disordered eating behaviors that have been reported to be associated with type 1 diabetes in adolescents and youth [46–48].

This systematic review shows different results regarding MNT and glycemic control. Few RCT demonstrated that a dietary intervention based on standard nutritional recommendations or healthy dietary pattern improved glycemic control in adolescents with type 1 diabetes without changes in insulin dose. However, other clinical trials did not find any effect of the dietary intervention on glycemic control. According to the published evidence, adolescents with type 1 diabetes who did not meet nutritional guidelines showed a poorer glycemic control due to a lower adherence to nutritional recommendations [49]. The Diabetes Control and Complications Trial observed that low adherence to a healthy eating regimen was associated with a poorer glycemic control and higher insulin dose in adults and youths with type 1 diabetes [50]. Furthermore, a cross-sectional study performed with adults and adolescents with type 1 diabetes observed that those participants with a higher adherence to the prescribed diet had an optimal glycemic control in comparison with a low-adherence group [51]. Maffeis et al. [52] observed a positive relationship between a saturated fatty acid-rich intake and a poorer glycemic control in adolescents with type 1 diabetes. Moreover, a retrospective longitudinal study performed to compare glycemic control and lipid profile of children and adolescents undergoing a carbohydrate-counting treatment with a dietary counseling based on caloric distribution of food, found an improvement of HbA1c with carbohydrate-counting diet [53]. According to the ADA recommendations, adolescents with type 1 diabetes should have a DSMES including MNT as part of diabetes care, with a physical activity program in addition of insulin therapy [2]. For this reason, the effectiveness of MNT on glycemic control and insulin dose of individuals with type 1 diabetes should be focused on overall lifestyle interventions, including physical activity.

Finally, only a few results have been published about MNT and cardiovascular risk factors such as BMI and lipid profile in adolescents with type 1 diabetes. In this review, results of the interventional studies are contradictory. A RCT demonstrated that a WPFD diet was not associated with BMI [32]; however, Dłużniak-Gołaska et al. [16] found that an interactive intervention was associated with an increased BMI. Furthermore, a WPFD diet was related to a lower total and HDL cholesterol [27]. Nevertheless, other clinical trials did not find any association between a dietary intervention and BMI or lipid profile [18, 38]. Dalsgaard et al. [53] did not observe significant changes in the lipid profile of the adolescents with type 1 diabetes with a carbohydrate-counting diet. However, in a cross-sectional study performed with adolescent-onset individuals with type 1 diabetes in Japan, a poorer lipid profile was associated with western dietary habits [54].

This systematic review provides an overview of the effectiveness of MNT in adolescents with type 1 diabetes. However, the search strategy did not include other databases apart from PubMed. This is the first review performed to assess whether MNT influences in the dietary pattern, diabetes self-management, glycemic control and cardiovascular risk factors. The methodology of this review following the Cochrane guidelines allows a critical assessment of the current scientific evidence. Moreover, the included studies with different target groups and interventions, with high and uncertain risk of bias should be considered as a weakness. For this reason, these findings could not be meta-analyzed. Finally, studies included in this review were only focused on dietary interventions without taking into account physical activity; thus, final conclusions could not be made about the potential effect of MNT as part of an integrated DSMES strategy.

In conclusion, few studies have demonstrated the potential effectiveness of MNT in adolescents with type 1 diabetes. Furthermore, these limited number of studies had a high risk of bias, precluding conclusions on this issue. Further research is needed to determine the effectiveness of MNT. In addition to MNT, physical activity should be included in future study interventions, which are two key components of the DSMES.

## Supplementary information

Table S1

Table S2

Table S3

Table S4
