# Supplementary material for: Effectiveness of medical nutrition therapy in adolescents with type 1 diabetes: a systematic review
Source: Nutr Diabetes. 2022 Apr 22;12:24. doi: 10.1038/s41387-022-00201-7 (PMC9033775; doi:10.1038/s41387-022-00201-7)
Supplement: Supplementary file 1 — Table S1 [file 41387_2022_201_MOESM1_ESM.docx]

**Table S1. Search strategy of the systematic review on December 2021**

| **Search** | **Query** | **Items found** |
| --- | --- | --- |
| 8 | Search ((((((((((("Diet"[Mesh] OR "Diet Therapy"[Mesh] OR "Feeding Behavior"[Mesh:NoExp] OR diet*[tiab] OR dietary habits[tiab] OR nutritional intake[tiab] OR nutri*[tiab] OR nutrient intake[tiab] OR eat*[tiab])))))) AND (("Diabetes Mellitus, Type 1"[Mesh:NoExp] OR type 1 diabetes[tiab] OR DM1[tiab] OR DMI[tiab])))))) AND (("Adolescent"[Mesh] OR adolescen*[tiab] OR teen*[tiab])))) | 2,378 |
| 7 | Search (((((((((("Diet"[Mesh] OR "Diet Therapy"[Mesh] OR "Feeding Behavior"[Mesh:NoExp] OR diet*[tiab] OR dietary habits[tiab] OR nutritional intake[tiab] OR nutri*[tiab] OR nutrient intake[tiab] OR eat*[tiab])))))) AND (("Diabetes Mellitus, Type 1"[Mesh:NoExp] OR type 1 diabetes[tiab] OR DM1[tiab] OR DMI[tiab])))))) AND (("Adolescent"[Mesh] OR adolescen*[tiab])) | 2,007 |
| 6 | Search (((((((("Diet"[Mesh] OR "Diet Therapy"[Mesh] OR "Feeding Behavior"[Mesh:NoExp] OR diet*[tiab] OR dietary habits[tiab] OR nutritional intake[tiab] OR nutri*[tiab] OR nutrient intake[tiab] OR eat*[tiab])))))) AND (("Diabetes Mellitus, Type 1"[Mesh:NoExp] OR type 1 diabetes[tiab] OR DM1[tiab] OR DMI[tiab])))) | 9,547 |
| 5 | Search ((((((("Diet"[Mesh] OR "Diet Therapy"[Mesh] OR "Feeding Behavior"[Mesh:NoExp] OR diet*[tiab] OR dietary habits[tiab] OR nutritional intake[tiab] OR nutri*[tiab] OR nutrient intake[tiab] OR eat*[tiab])))))) AND (("Diabetes Mellitus, Type 1"[Mesh:NoExp] OR type 1 diabetes[tiab])) | 6,894 |
| 4 | Search ((((("Diet"[Mesh] OR "Diet Therapy"[Mesh] OR "Feeding Behavior"[Mesh:NoExp] OR diet*[tiab] OR dietary habits[tiab] OR nutritional intake[tiab] OR nutri*[tiab] OR nutrient intake[tiab] OR eat*[tiab])))) | 1,131,010 |
| 3 | Search (((("Diet"[Mesh] OR "Diet Therapy"[Mesh] OR "Feeding Behavior"[Mesh:NoExp] OR diet*[tiab] OR dietary habits[tiab] OR nutritional intake[tiab] OR nutri*[tiab] OR nutrient intake[tiab]))) | 1,119,576 |
| 2 | Search ((("Diet"[Mesh] OR "Diet Therapy"[Mesh] OR "Feeding Behavior"[Mesh:NoExp] OR diet*[tiab] OR dietary habits[tiab] OR nutritional intake[tiab])) | 793,698 |
| 1 | Search (("Diet"[Mesh] OR "Diet Therapy"[Mesh] OR "Feeding Behavior"[Mesh:NoExp]) | 377,466 |

**MEDLINE (PubMed)**

**EMBASE (embase.com)**

| **Search** | **Query** | **Items found** |
| --- | --- | --- |
| 20 | #7 AND #12 AND #18 AND [embase]/lim | 2,999 |
| 19 | #7 AND #12 AND #18 | 3,714 |
| 18 | #13 OR #14 OR #15 OR #16 OR #17 | 2,377,893 |
| 17 | teen*:ti AND ([embase]/lim OR [medline]/lim) | 15,286 |
| 16 | children:ti AND ([embase]/lim OR [medline]/lim) | 727,045 |
| 15 | youth:ti AND ([embase]/lim OR [medline]/lim) | 37,063 |
| 14 | adolescent*:ti,ab AND ([embase]/lim OR [medline]/lim) | 370,388 |
| 13 | 'adolescent'/exp AND ([embase]/lim OR [medline]/lim) | 1,786,782 |
| 12 | #8 OR #9 OR #10 OR #11 | 155,319 |
| 11 | (('type 1' NEAR/5 diabetes):ti,ab) AND ([embase]/lim OR [medline]/lim) | 80,104 |
| 10 | iddm:ti,ab AND ([embase]/lim OR [medline]/lim) | 7,921 |
| 9 | 'insulin dependent diabetes mellitus':ti,ab AND ([embase]/lim OR [medline]/lim) | 17,929 |
| 8 | 'insulin dependent diabetes mellitus'/exp AND ([embase]/lim OR [medline]/lim) | 130,254 |
| 7 | #1 OR #2 OR #3 OR #4 OR #5 OR #6 | 1,483,869 |
| 6 | ((carbohydrate NEXT/1 counting):ti) AND ([embase]/lim OR [medline]/lim) | 269 |
| 5 | diet*:ti,ab AND ([embase]/lim OR [medline]/lim) | 789,964 |
| 4 | nutrition*:ti,ab AND ([embase]/lim OR [medline]/lim) | 409,476 |
| 3 | 'feeding behavior'/exp AND ([embase]/lim OR [medline]/lim) | 193,518 |
| 2 | 'diet therapy'/exp AND ([embase]/lim OR [medline]/lim) | 392,291 |
| 1 | 'diet'/exp AND ([embase]/lim OR [medline]/lim) | 383,334 |
