# Supplementary material for: Effectiveness of medical nutrition therapy in adolescents with type 1 diabetes: a systematic review
Source: Nutr Diabetes. 2022 Apr 22;12:24. doi: 10.1038/s41387-022-00201-7 (PMC9033775; doi:10.1038/s41387-022-00201-7)
Supplement: Supplementary file 2 — Table S2 [file 41387_2022_201_MOESM2_ESM.docx]

|  | **Dłużniak-Gołaska 2019**^1^ | **Nansel 2015**^2^ | **Gökşen 2014**^3^ | **Spiegel 2012**^4^ |
| --- | --- | --- | --- | --- |
| **RANDOM SEQUENCE GENERATION**  (selection bias) | low - randomization was generated using a computer software. | low - permuted block randomization scheme. | uncertain - this data was not described in detail. | low - randomization was generated using a computer software. |
| **ALLOCATION CONCEALMENT**  (selection bias) | high - allocation was performed by the trainer. | low - group assignment was performed by an online data management system. | uncertain - this data was not described in detail. | uncertain - this data was not described in detail. |
| **BLINDING OF PARTICIPANTS AND PERSONNEL**  (performance bias) | uncertain - patients were not informed which group they joined (single-blind). | high - participants were informed of their group assignment at the second study visit. | high - the study probably had an open design. | high - the study probably had an open design. |
| **BLINDING OF OUTCOME ASSESSMENT**  (detection bias) | uncertain - this data was not described in detail. | uncertain - this data was not described in detail. | high - the study probably had an open design. | high - the study probably had an open design. |
| **INCOMPLETE OUTCOME DATA** (attrition bias) | low - sample size calculation accounted a larger number of participants to account a proportion of losses of follow-up. | high - unequal attrition between study groups. Authors recognize the possible sampling bias due to the withdrawal in the intervention group. | uncertain - this data was not described in detail. | low - sample size calculation accounted a larger number of participants to account a proportion of losses of follow-up. |
| **SELECTIVE REPORTING**  (reporting bias) | uncertain - study protocol was not available. | low - the study analyzed, and published results specified in the study protocol. | uncertain - study protocol was not available. | uncertain - study protocol was not available. |

**Table S2. Risk of bias assessment of the randomized clinical trials included in the systematic review**

|  | **Marquard 2011**^5^ | **Gilbertson 2001**^6^ | **Donaghue 2000**^7^ | **Pichert 1994**^8^ | **Hackett 1989**^9^ |
| --- | --- | --- | --- | --- | --- |
| **RANDOM SEQUENCE GENERATION**  (selection bias) | high – randomization was performed with a minimization according to age, gender, BMI, duration of diabetes and glycemic control | low - randomization was generated using a computer software. | uncertain - this data was not described in detail. | uncertain - this data was not described in detail. | uncertain - this data was not described in detail. |
| **ALLOCATION CONCEALMENT**  (selection bias) | high – the study had an open-label design | uncertain - although the researchers generated a random number table, they described that the blocks of participants were assigned consecutively. | uncertain - this data was not described in detail. | uncertain - this data was not described in detail. | uncertain - this data was not described in detail. |
| **BLINDING OF PARTICIPANTS AND PERSONNEL**  (performance bias) | high – the study had an open-label design | high - the study probably had an open design. | uncertain - this data was not described in detail. | uncertain - this data was not described in detail. | uncertain - this data was not described in detail. |
| **BLINDING OF OUTCOME ASSESSMENT**  (detection bias) | high - food diaries were analyzed by the researcher. | high - food diaries were analyzed by the researcher. | high - the study probably had an open design. | high - the study probably had an open design. | high - the study probably had an open design. |
| **INCOMPLETE OUTCOME DATA**  (attrition bias) | low - study groups were equality without significant follow-up withdrawn. | high - unequal attrition between study groups. | low - study groups were equality without significant follow-up withdrawn. | uncertain - this data was not described in detail. | uncertain - this data was not described in detail. |
| **SELECTIVE REPORTING** (reporting bias) | uncertain - study protocol was not available. | uncertain - study protocol was not available. | uncertain - study protocol was not available. | uncertain - study protocol was not available. | uncertain - study protocol was not available. |

**REFERENCES**

1 . Dłużniak-Gołaska K, Panczyk M, Szostak-Węgierek D, Szypowska A, Sińska B. Analysis of the diet quality and dietary habits of children and adolescents with type 1 diabetes. *Diabetes, Metab. Syndr. Obes. Targets Ther.* **12**, 161–170 (2019).

2 . Nansel TR, Laffel LMB, Haynie DL, Mehta SN, Lipsky LM, Volkening LK *et al.* Improving dietary quality in youth with type 1 diabetes: Randomized clinical trial of a family-based behavioral intervention. *Int. J. Behav. Nutr. Phys. Act.* **12,** 1–11 (2015).

3 . Gökşen D, Altinok YA, Özen S, Demir G, Darcan Ş. Effects of carbohydrate counting method on metabolic control in children with type 1 diabetes mellitus. *J. Clin. Res. Pediatr. Endocrinol.* **6**, 74–78 (2014).

4 . Spiegel G, Bortsov A, Bishop FK, Owen D, Klingensmith GJ, Mayer-Davis EJ *et al.* Randomized Nutrition Education Intervention to Improve Carbohydrate Counting in Adolescents with Type 1 Diabetes Study: Is More Intensive Education Needed? *J. Acad. Nutr. Diet.* **112**, 1736–1746 (2012).

5 . Marquard J, Stahl A, Lerch C, Wolters M, Grotzke-Leweling M, Mayatepek E *et al.* A prospective clinical pilot-trial comparing the effect of an optimized mixed diet versus a flexible low-glycemic index diet on nutrient intake and HbA1c levels in children with type 1 diabetes. *J. Pediatr. Endocrinol. Metab.* **24**, 441–447 (2011).

6 . Gilbertson HR, Evans S, Brand-Miller JC, Chondros P, Thorburn AW, Werther GA. The Effect of Flexible Low Glycemic Diets on Glycemic Control in Children With Type 1 Diabetes. *Diabetes Care*. **24**, 1137–43 (2001).

7 . Donaghue KC, Pena MM, Chan AKF, Blades BL, King J, Storlien LH *et al.* Beneficial effects of increasing monounsaturated fat intake in adolescents with type 1 diabetes. *Diabetes Res. Clin. Pract.* **48**, 193–199 (2000).

8 . Pichert JW, Smeltzer C, Snyder GM, Gregory RP, Smeltzer R, Kinzer CK. Traditional vs Anchored Instruction for Diabetes-Related Nutritional Knowledge, Skills, and Behavior. *Diabetes Educ.* **20**, 45–48 (1994).

9 . Hackett AF, Court S, Matthews JNS, McCowen C, Parkin JM. Do education groups help diabetics and their parents? *Arch. Dis. Child.* **64,** 977–1003 (1989).
