# Supplementary material for: Effectiveness of medical nutrition therapy in adolescents with type 1 diabetes: a systematic review
Source: Nutr Diabetes. 2022 Apr 22;12:24. doi: 10.1038/s41387-022-00201-7 (PMC9033775; doi:10.1038/s41387-022-00201-7)
Supplement: Supplementary file 3 — Table S3 [file 41387_2022_201_MOESM3_ESM.docx]

**Table S3. Risk of bias assessment of the pre-post intervention studies included in the systematic review**

|  | **Marigliano 2013**^1^ | **Cadario 2012**^2^ | **Lorini 1990**^3^ |
| --- | --- | --- | --- |
| **CONFOUNDING**  (baseline impact from prognostic factors) | high – analyses were conducted without any adjustment to confounding factors | high – analyses were conducted without any adjustment to confounding factors | high – analyses were conducted without any adjustment to confounding factors |
| **CONFOUNDING**  (of intervention) | low – intervention according ADA and ISPAD guidelines | low – intervention according Scientific Society guidelines | uncertain – the intervention was standardised but was not based on recommendations from guidelines |
| **SELECTION BIAS**  (study simple, exclusions during eligibility) | uncertain – the researchers probably included people with a controlled disease, as they implemented strict inclusion criteria excluding co morbidities | low – researchers included consecutive patients attending a clinic in a defined period of time | uncertain – the researchers did not describe their inclusion criteria |
| **BIAS IN MEASUREMENT OF INTERVENTIONS** | uncertain – intervention according ADA and ISPAD guidelines, but compliance with dietary records was not measured and depends on differences in health literacy. | uncertain – standardised intervention, but compliance with dietary records was not measured and depends on differences in health literacy. | uncertain – the researchers did not describe details on how the intervention was monitored |
| **BIAS DUE TO DEVIATIONS FROM INTENDED INTERVENTIONS** | uncertain – adherence / compliance could not be warranted in all the participants, with anticipated differences in health literacy, and it was not measured. | uncertain – adherence / compliance could not be warranted in all the participants, with anticipated differences in health literacy, and it was not measured. Patient were permitted to practice physical activity that was not evaluated (researchers reported that any patient performed intensive exercise) | uncertain –not described |
| **BIAS IN OUTCOME MEASUREMENT** | low – participants were trained to complete food and dietary records | low – participants were trained to complete food and dietary records | uncertain –not described |
| **MISSING DATA** | low – the researchers did not describe any loss from follow up | low – lower analysed patients than described at inclusion, without a description of reasons to their withdrawal or loss from follow up | uncertain –not described |

**REFERENCES**

1 . Marigliano M, Morandi A, Maschio M, Sabbion A, Contreas G, Tomasselli F *et al.* Nutritional education and carbohydrate counting in children with type 1 diabetes treated with continuous subcutaneous insulin infusion: The effects on dietary habits, body composition and glycometabolic control. *Acta Diabetol.* **50**, 959–964 (2013).

2 . Cadario F, Prodam F, Pasqualicchio S, Bellone S, Bonsignori I, Demarchi I *et al.* Lipid profile and nutritional intake in children and adolescents with Type 1 diabetes improve after a structured dietician training to a Mediterranean-style diet. *J. Endocrinol. Invest.* **35**, 160–168 (2012).

3 . Lorini R, Ciriaco O, Salvatoni A, Livieri C, Larizza D, D’Annunzio G. The influence of dietary education in diabetic children. *Diabetes Res. Clin. Pract.* **9**, 279–285 (1990).
