# Supplementary material for: Effectiveness of medical nutrition therapy in adolescents with type 1 diabetes: a systematic review
Source: Nutr Diabetes. 2022 Apr 22;12:24. doi: 10.1038/s41387-022-00201-7 (PMC9033775; doi:10.1038/s41387-022-00201-7)
Supplement: Supplementary file 4 — Table S4 [file 41387_2022_201_MOESM4_ESM.docx]

**Table S4. Studies included in the systematic review and their reports**

| **ID** | **REFERENCES** |
| --- | --- |
| **NCT00999375**  RCT | **Sanjeevi 2019**  Sanjeevi N, Lipsky L, Liu A, Nansel T. Differential reporting of fruit and vegetable intake among youth in a randomized controlled trial of a behavioural nutrition intervention. Int J Behav Nutr Phys Act. 2019 Feb 1;16(1):15.  **Lipsky 2019**  Lipsky LM, Haynie DL, Liu A, Nansel TR. Resemblance of Diet Quality in Families of Youth with Type 1 Diabetes Participating in a Randomized Controlled Behavioral Nutrition Intervention Trial in Boston, MA (2010-2013): A Secondary Data Analysis. J Acad Nutr Diet. 2019 Jan;119(1):98-105. doi: 10.1016/j.jand.2018.07.025.  **Sanjeevi 2018**  Sanjeevi N, Lipsky LM, Nansel TR. Cardiovascular Biomarkers in Association with Dietary Intake in a Longitudinal Study of Youth with Type 1 Diabetes. Nutrients. 2018 Oct 19;10(10). pii: E1552.  **Nansel 2018**  Nansel TR, Lipsky LM, Haynie DL, Eisenberg MH, Dempster K, Liu A. Picky Eaters Improved Diet Quality in a Randomized Behavioral Intervention Trial in Youth with Type 1 Diabetes. J Acad Nutr Diet. 2018 Feb;118(2):308-316.  **Eisenberg 2018**  Eisenberg Colman MH, Quick VM, Lipsky LM, Dempster KW, Liu A, Laffel LMB, Mehta SN, Nansel TR. Disordered Eating Behaviors Are Not Increased by an Intervention to Improve Diet Quality but Are Associated With Poorer Glycemic Control Among Youth With Type 1 Diabetes. Diabetes Care. 2018 Apr;41(4):869-875.  **Eisenberg 2017**  Eisenberg MH, Lipsky LM, Gee B, Liu A, Nansel TR. Parent healthful eating attitudes and motivation are prospectively associated with dietary quality among youth with type 1 diabetes. Vulnerable Child Youth Stud. 2017;12(3):226-240.  **Lipsky 2017**  Lipsky LM, Gee B, Liu A, Nansel TR. Body mass index and adiposity indicators associated with cardiovascular biomarkers in youth with type 1 diabetes followed prospectively. Pediatr Obes. 2017 Dec;12(6):468-476.  **Nansel 2016a**  Nansel TR, Lipsky LM, Liu A. Greater diet quality is associated with more optimal glycemic control in a longitudinal study of youth with type 1 diabetes. Am J Clin Nutr. 2016 Jul;104(1):81-7.  **Nansel 2016b**  Nansel TR, Lipsky LM, Eisenberg MH, Liu A, Mehta SN, Laffel LM. Can Families Eat Better Without Spending More? Improving Diet Quality Does Not Increase Diet Cost in a Randomized Clinical Trial among Youth with Type 1 Diabetes and Their Parents. J Acad Nutr Diet. 2016 Nov;116(11):1751-1759.e1.  **Lipsky 2016**  Lipsky LM, Gee B, Liu A, Nansel TR. Glycemic control and variability in association with body mass index and body composition over 18months in youth with type 1 diabetes. Diabetes Res Clin Pract. 2016 Oct;120:97-103.  **Nansel 2015**  Nansel TR, Laffel LM, Haynie DL, Mehta SN, Lipsky LM, Volkening LK, Butler DA, Higgins LA, Liu A. Improving dietary quality in youth with type 1 diabetes: randomized clinical trial of a family-based behavioral intervention. Int J Behav Nutr Phys Act. 2015 May 8;12:58. |
| **Dłużniak-Gołaska**  **2019**  RCT | **Dłużniak-Gołaska 2019**  Dłużniak-Gołaska K, Panczyk M, Szypowska A, Sińska B, Szostak-Węgierek D. Interactive Nutrition Education Is More Effective in Terms of Improved Levels of Glycated Hemoglobin in Adolescent Patients with Poorly Controlled Type 1 Diabetes - A Randomized Study. Diabetes Metab Syndr Obes. 2019 Dec 10;12:2619-2631. |
| **Gökşen 2014**  RCT | **Gökşen 2014**  Gökşen D, Atik Altınok Y, Ozen S, Demir G, Darcan S. Effects of carbohydrate counting method on metabolic control in children with type 1 diabetes mellitus. J Clin Res Pediatr Endocrinol. 2014;6(2):74-8. doi: 10.4274/Jcrpe.1191. |
| **CCAT study**  RCT | **Spiegel 2012**  Spiegel G, Bortsov A, Bishop FK, Owen D, Klingensmith GJ, Mayer-Davis EJ, Maahs DM. Randomized nutrition education intervention to improve carbohydrate counting in adolescents with type 1 diabetes study: is more intensive education needed? J Acad Nutr Diet. 2012 Nov;112(11):1736-46. |
| **Marquard 2011**  RCT | **Marquard 2011**  Marquard J, Stahl A, Lerch C, Wolters M, Grotzke-Leweling M, Mayatepek E, Meissner T. A prospective clinical pilot-trial comparing the effect of an optimized mixed diet versus a flexible low-glycemic index diet on nutrient intake and HbA(1c) levels in children with type 1 diabetes. J Pediatr Endocrinol Metab. 2011;24(7-8):441-7. |
| **Gilbertson 2001**  RCT | **Gilbertson 2003**  Gilbertson HR, Thorburn AW, Brand-Miller JC, Chondros P, Werther GA. Effect of low-glycemic-index dietary advice on dietary quality and food choice in children with type 1 diabetes. Am J Clin Nutr. 2003 Jan;77(1):83-90.  **Gilbertson 2001**  Gilbertson HR, Brand-Miller JC, Thorburn AW, Evans S, Chondros P, Werther GA. The effect of flexible low glycemic index dietary advice versus measured  carbohydrate exchange diets on glycemic control in children with type 1 diabetes. Diabetes Care. 2001 Jul;24(7):1137-43. |
| **Donaghue 2000**  RCT | **Donaghue 2000**  Donaghue KC, Pena MM, Chan AK, Blades BL, King J, Storlien LH, Silink M. Beneficial effects of increasing monounsaturated fat intake in adolescents with type 1 diabetes. Diabetes Res Clin Pract. 2000 Jun;48(3):193-9. |
| **Pichert 1994**  RCT | **Pichert 1994**  Pichert JW, Smeltzer C, Snyder GM, Gregory RP, Smeltzer R, Kinzer CK. Traditional vs anchored instruction for diabetes-related nutritional knowledge, skills, and behavior. Diabetes Educ. 1994 Jan-Feb;20(1):45-8. |
| **Hackett 1989**  RCT | **Hackett 1989**  Hackett AF, Court S, Matthews JN, McCowen C, Parkin JM. Do education groups help diabetics and their parents? Arch Dis Child. 1989 Jul;64(7):997-1003. |
| **Marigliano 2013**  Interventional, uncontrolled, prospective cohort | **Marigliano 2013**  Marigliano M, Morandi A, Maschio M, Sabbion A, Contreas G, Tomasselli F, Tommasi M, Maffeis C. Nutritional education and carbohydrate counting in children with type 1 diabetes treated with continuous subcutaneous insulin infusion: the effects on dietary habits, body composition and glycometabolic control. Acta Diabetol. 2013 Dec;50(6):959-64. |
| **Cadario 2012**  Interventional, uncontrolled, prospective cohort | **Cadario 2012**  Cadario F, Prodam F, Pasqualicchio S, Bellone S, Bonsignori I, Demarchi I, Monzani A, Bona G. Lipid profile and nutritional intake in children and adolescents with Type 1 diabetes improve after a structured dietician training to a Mediterranean-style diet. J Endocrinol Invest. 2012 Feb;35(2):160-8. |
| **Lorini 1990**  Interventional, uncontrolled, prospective cohort | **Lorini 1990**  Lorini R, Ciriaco O, Salvatoni A, Livieri C, Larizza D, D'Annunzio G. The influence of dietary education in diabetic children. Diabetes Res Clin Pract. 1990 Jul;9(3):279-85. |
